# Evaluation of Current Therapies for COVID-19 Treatment

**DOI:** 10.3390/microorganisms8081097

**Published:** 2020-07-22

**Authors:** Atin Sethi, Horacio Bach

**Affiliations:** 1 Department of Medicine, University of British Columbia, Vancouver, BC V6T 1Z3, Canada; atinsethi@alumni.ubc.ca; 2 Department of Medicine, Division of Infectious Diseases, University of British Columbia, Vancouver, BC ADD ED8, Canada

**Keywords:** COVID-19, SARS-CoV-2, SARS-CoV, MERS, therapies, antivirals, clinical trials

## Abstract

The virus SARS-CoV-2, the etiological agent of COVID-19, is responsible for more than 400,000 deaths worldwide as of 10 June 2020. As a result of its recent appearance (December 2019), an efficacious treatment is not yet available. Although considered a lung infection since its emergence, COVID-19 is now causing multiple organ failure, requiring a continuous adjustment in the procedures. In this review, we summarize the current literature surrounding unproven therapies for COVID-19. Analyses of the clinical trials were grouped as chemotherapy, serotherapy, anticoagulant, and the use of human recombinant soluble ACE2 therapies. We conclude that, while no agent has hit the threshold for quality of evidence to demonstrate efficacy and safety, preliminary data show potential benefits. Moreover, there is a possibility for harm with these unproven therapies, and the decision to treat should be based on a comprehensive risk–benefit analysis.

## 1. Introduction

The coronavirus SARS-CoV-2 the etiological agent of COVID-19, emerged in Wuhan, China, in December 2019 [1]. Since then, the disease has disseminated globally with a death toll of more than 400,000 deaths worldwide as of June 2020 and has been declared as a pandemic by the WHO. 

After the first cases of COVID-19 emerged, the disease was characterized as pneumonia, which could progress to severe illness, leading to death. After the number of COVID-19 cases increased, and more information was collected, the disease is now considered systemic and characterized by a multi-organ failure [2,3]. 

Although many therapies have been implemented, there are no currently well-researched and approved therapies for the treatment of COVID-19 or other illnesses caused by coronaviruses. While the FDA has approved hydroxychloroquine for emergency use, it urges that practicing evidence-based medicine remains essential [4].

Testing previously existing therapeutics on a compassionate use or emergency use basis should only be done when necessary. There may be a real potential for harm to the patient if therapies are used without caution. In some cases, the risk of harm may be balanced with benefits, but it is not in many cases. However, the search for a safe and efficacious therapeutic for the treatment of COVID-19 should be the highest priority for stakeholders. The scientific community’s enormous effort has been shown through numerous publications and the initiation of many high-quality randomized controlled trials. 

**Figure 1:**
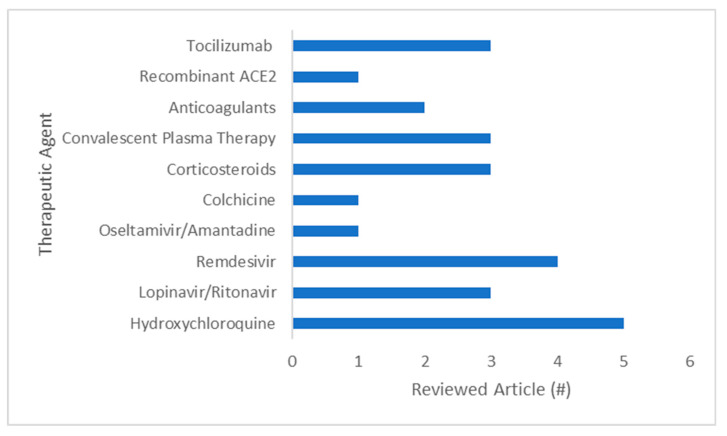
Number of publications reviewed in this review.

In this review, we compiled and analyzed the current evidence on therapeutics under investigation for COVID-19, using the following keywords: COVID-19, SARS-CoV-2, SARS, MERS, hydroxychloroquine, azithromycin, lopinavir, ritonavir, remdesivir, oseltamivir, amantadine, colchicine, corticosteroids, convalescent plasma, anticoagulation, mortality, recombinant, angiotensin, tocilizumab, and ACE2. Medline, Medrxiv, PubMed, and clinicaltrials.gov were searched. The total number of studies found was 20 (Figure 1), grouped into chemotherapy, serotherapy, anticoagulant, and human recombinant soluble ACE2 therapies (Table 1).

## 2. Chemotherapies

### 2.1. Hydroxychloroquine ± Azithromycin

Hydroxychloroquine is primarily used in the treatment of malaria and rheumatoid arthritis [31,32]. The mechanism of action of hydroxychloroquine has not been elucidated with confidence, and only in vitro studies at a cellular level are available. The drug is thought to exert its effect through suppression of antigen processing by disrupting the loading of Major Histocompatibility Complex II (MHCII) with antigen inside cells, interfering with the lysosome and endosome function by increasing the pH [33,34]. This reduction of MHCII theoretically decreases T-cell activation [35]. Moreover, autophagy is another process that appears to be regulated by hydroxychloroquine [36]. In the context of SARS-CoV-2, the exact mechanism has not been elucidated. It is reasonable to suggest that the anti-inflammatory effects of the drugs may theoretically provide an effect on COVID-19 disease progression.

The use of hydroxychloroquine has to be under strict medical surveillance because the adverse effects include ocular toxicity, gastrointestinal discomfort, rash, and nightmares [37].

Azithromycin, a derivative of erythromycin, is a macrolide class antibiotic [38] that inhibits the 50 S subunit of bacterial ribosomes, thereby inhibiting protein synthesis [39]. A mechanism of action against SARS-CoV-2 has not been identified. The drug is known to cause gastrointestinal discomfort. When combined with hydroxychloroquine, an increase in time from ventricular depolarization to repolarization (QT interval prolongation) has been observed [9].

A description of the clinical trials (Table 1) related to hydroxychloroquine alone or azithromycin is described.

#### 2.1.1. Efficacy of Hydroxychloroquine in a Randomized Clinical Trial

This clinical trial is a double-blind, randomized control clinical trial centered at the Renmin Hospital in Wuhan, China (February 4–28, 2020). The study includes 62 adults >18 years old (mean 44.7) with a positive COVID-19 diagnosis using PCR, pneumonia on chest CT, and mild disease (SaO_2_/SpO_2_ >93%, PaO_2_/FIO_2_ >300). Endpoints included time to clinical recovery (TTCR), clinical characteristics, and chest CT findings. Improvement was observed in TTCR in the hydroxychloroquine/azithromycin group compared to control: fever (2.2 vs. 3.2 days) and cough remission (2.0 vs. 3.1 days). Pneumonia improved in 25/32 (78%) of the patients treated with hydroxychloroquine/azithromycin and 17/32 (53%) in the control group. Four patients in the control group progressed to severe illness, and two patients developed mild side effects related to hydroxychloroquine. Limitations for this study include that the findings are not generalizable to those with severe disease, the absence of time of positive to negative conversion in test results, and the relatively small sample size.

#### 2.1.2. Hydroxychloroquine and Azithromycin as a Treatment of COVID-19 in a Non-Randomized Clinical Trial

This preliminary trial (*n* = 36) [6] showed a large reduction in viral carriage (57.1% test negative vs. 12.5%) in those patients assigned with 600 mg hydroxychloroquine alone (*n* = 14) per day vs. control (*n* = 16). Of those who received hydroxychloroquine/azithromycin (*n* = 6), 100% of the patients tested negative by nasopharyngeal PCR. The study is limited by its small sample size.

#### 2.1.3. Clinical and Microbiological Effects of a Combination of Hydroxychloroquine and Azithromycin in 80 COVID-19 Patients with at Least a Six-Day Follow-Up: An Observational Study

This study was conducted at a single hospital where 80 patients with no contraindications received hydroxychloroquine/azithromycin. The treatment was discontinued if QTc >500 ms. A PCR value <34 cycles and positive cultures were considered potentially contagious. More than half of the patients (53%) had chronic comorbid conditions such as hypertension and diabetes. The mean PCR cycle threshold value was 23.4.

The study was divided into two groups of patients, those with primarily upper and lower respiratory tract symptoms. Endpoints included supplemental oxygen requirement and transfer to ICU, contagiousness assessed by PCR and culture, and length of stay in hospital. Adverse events were rare and minor. Overall, 81.3% of the patients were discharged with a favorable outcome, whereas 15% required oxygen therapy. Three were transferred to ICU, two improved, and one remained in ICU. One death was recorded.

Ninety-three percent of patients had a negative PCR at Day 8, whereas all patients were negative by Day 12. Viral cultures were negative in 97.5% of patients by Day 5. Of the 65 patients discharged, the mean time (treatment initiation to discharge) was 4.1 days. This study demonstrates the possible role of hydroxychloroquine/azithromycin in decreasing the duration of viral infection. Limitations include the absence of randomization, blinding, or control group. The trial relies on a preclinical study to demonstrate superiority to control. Viral cultures were not provided with a control; therefore, it is not possible to establish efficacy or effect size for that outcome. The time from initiation to discharge also requires control to show efficacy.

#### 2.1.4. No Evidence of Rapid Antiviral Clearance or Clinical Benefit with the Combination of Hydroxychloroquine and Azithromycin in Patients with Severe COVID-19 Infection

This study includes 11 patients with numerous severe comorbidities. At treatment initiation, 10/11 (91%) patients had fever and required nasal oxygen therapy. Within five days, one patient died, two were transferred to ICU, and hydroxychloroquine and azithromycin were discontinued in one patient due to QTc prolongation [8].

The presence of the virus was conducted using nasal swabs in 10 patients using PCR. Eight of ten (80%) patients were still positive for the virus’s presence at Days 5-6 post-treatment. The author suggests that there is no benefit from hydroxychloroquine/azithromycin treatment. However, the study is methodologically flawed (no control, blinding, or randomization) and is not sufficiently powered to support such a claim.

#### 2.1.5. The QT Interval in Patients with SARS-CoV-2 Infection Treated with Hydroxychloroquine/Azithromycin

This retrospective study demonstrates the real risk of prolonged QT interval in patients (*n* = 84) treated with hydroxychloroquine and azithromycin for COVID-19 [9]. After the treatment with hydroxychloroquine/azithromycin, 11% of the patients developed a QTc >500 ms, which presented a high risk for arrhythmia, whereas 30% showed a QTc increase more significant than 40 ms. The development of acute renal failure was found to be a strong predictor of extreme QTc prolongation. Although the focus of this study was not to explore the efficacy of hydroxychloroquine/azithromycin, it outlines the importance of appropriate risk–benefit analysis while treating patients with COVID-19. While this study observed prolonged QTc, it would be of clinical interest to note the incidence of a life-threatening arrhythmia (if present) in this population.

## 3. Antiviral Therapies

### 3.1. Lopinavir/Ritonivir

Lopinavir is used as a retroviral therapy to exert an anti-HIV effect through HIV-1 viral protease inhibition, whereas ritonavir boosts the effects of lopinavir [40]. In the context of coronaviruses, the 3CL protease has been a target of interest due to its function in liberating individual proteins from the unprocessed polyprotein. Lopinavir is known to cause gastrointestinal discomfort, serum lipid elevations, and has many drug–drug interactions that may result in hepatotoxicity, hemophilia, metabolic derangement, and heart block [41,42,43]. Adverse effects of ritonavir include paresthesia, hepatitis, diarrhea, and nausea. The combination has been used for the treatment of MERS through its activity on the 3CL protease in SARS-CoV, but no high-quality data on its efficacy have been published [44]. The following studies have been published (Table 1).

#### 3.1.1. A Trial of Lopinavir–Ritonavir in Adults Hospitalized with Severe COVID-19

This randomized control trial [10] of 199 patients explored the efficacy of lopinavir–ritonavir in hospitalized COVID-19 patients with relatively mild respiratory illness. The authors demonstrated that there is no significant improvement in time to clinical improvement or mortality compared to patients who received standard care. There was also no significant difference in detectable viral RNA between the groups. Adverse effects were noted in the lopinavir–ritonavir group (13.8% stopped early), although serious adverse events were frequent in the standard care group. The authors concluded that there was no benefit to the use of lopinavir/ritonavir beyond standard care. While this is one of the more extensive studies of COVID-19 therapies, the sample size is not ideal. Further, little or no information was given on patient comorbidities.

#### 3.1.2. An Exploratory Randomized, Controlled Study on the Efficacy and Safety of Lopinavir/Ritonavir or Arbidol Treating Adult Patients Hospitalized with Mild/Moderate COVID-19 (ELACOI)

This exploratory randomized control trial included 44 patients (21 lopinavir/ritonavir, 16 arbidol, and 7 neither). Similar baseline characteristics were observed in patients. There was no statistical difference found in time for positive to negative conversion of the viral RNA test between treatment groups. Five (23.8%) patients from the lopinavir/ritonavir group experienced adverse events. No adverse events were reported in the other groups. Limitations include the small sample size and imbalanced groups. The study had intended to recruit more patients but was unable to.

#### 3.1.3. Factors Associated with Prolonged Viral Shedding and Impact of Lopinavir/Ritonavir Treatment in Patients with SARS-CoV-2 Infection

This retrospective study included all test-positive cases (*n* = 120) at a single site in Wuhan, China [11]. Seventy-eight patients received lopinavir/ritonavir, and 42 did not. The authors demonstrated a significant increase in the duration of viral shedding in those who did not receive lopinavir/ritonavir (OR 2.42; 1.10–5.36). There was a positive correlation between age and length of viral shedding. Comorbidities or systemic corticosteroid use were not associated with changes in the duration of viral shedding. Limitations include retrospective design and small sample size.

### 3.2. Remdesivir

Remdesivir is an adenosine nucleotide analog that interferes with the function of the viral RNA dependent RNA polymerase [45]. The drug is still in its experimental stage, and a robust side effect profile has not been published. The following studies were published (Table 1).

#### 3.2.1. Remdesivir and Chloroquine Effectively Inhibit the Recently Emerged Novel Coronavirus (2019-nCoV) in Vitro

An in vitro study demonstrated inhibition of SARS-CoV-2 with the use of remdesivir and chloroquine [13]. The results of the study suggest a recommendation for human trials using these agents. For example, the effective concentrations (EC) were 1.76 µmol/L for EC_90_ in non-human primates for remdesivir and 6.09 µmol/L for EC_90_ in non-human primates for chloroquine.

#### 3.2.2. Prophylactic and Therapeutic Remdesivir (GS-5734) Treatment in the Rhesus Macaque Model of MERS-CoV Infection

This study is shown here because remdesivir was used with success in the treatment of MERS-CoV infection [14]. The possible prophylactic and therapeutic efficacy of remdesivir was established in this controlled trial through the inoculation of 18 Rhesus macaques. Remdesivir administration 24 h before the inoculation of the virus led to complete prevention of infection. In addition, remdesivir administration 12 h post-inoculation demonstrated a clear clinical benefit, including a reduction in clinical signs, reduced viral replication in the lungs, and decreased number and severity of lung lesions. Further clinical studies are necessary to demonstrate the efficacy of the drug as a therapy in COVID-19.

#### 3.2.3. First Case of 2019 Novel Coronavirus in the United States

This case report demonstrates the potential role that remdesivir may have played in the improvement of clinical status. The condition of the patient in question declined for seven days in the hospital, and remdesivir was used as a therapy [15]. The following day, the patient’s condition improved; supplemental oxygen was discontinued, and oxygen saturation rose to 94–96%. Rales were no longer heard; the patient became afebrile and had an improvement in cough. It is not reasonable to make conclusions from a single case, but further investigation is warranted.

#### 3.2.4. First 12 Patients with Coronavirus Disease 2019 (COVID-19) in the United States

In this study, 3 of 12 patients received remdesivir under compassionate use [16]. Mild GI symptoms were noted, as well as transient aminotransferase elevation. One episode of bloody stool was noted. All patients recovered or improved. The authors could not comment on efficacy, as this was not a randomized control trial to investigate the drug.

#### 3.2.5. Adaptive COVID-19 Treatment Trial (ACTT)

There is currently a lack of randomized controlled trials on the therapeutic effects of remdesivir for coronavirus and coronavirus-like illnesses. ACTT is a multi-center, randomized, double blind, placebo-control trial that is setting out to evaluate the efficacy and safety of remdesivir [17]. The estimated enrollment is 440 participants, and the study has posted an estimated completion date of April 2023.

### 3.3. Oseltamivir and Amantadine

Oseltamivir is an anti-influenza drug that exerts its effect through neuraminidase inhibition [46]. Adverse effects of oseltamivir include nausea, vomiting, and abdominal pain. Amantadine is active against influenza A and exerts its effect through blockade of the viral M2 proton ion channel [47]. Adverse effects of amantadine include nausea, anorexia, and CNS toxicity (nervousness, insomnia, and light-headedness).

#### Inhibition of SARS Coronavirus Infection in Vitro with Clinically Approved Antiviral Drugs

Currently, there is no evidence of in vitro inhibition of SARS-CoV-2 with oseltamivir and amantadine. The inhibition of the cytopathic effects of SARS-CoV-2 was observed when combined interferons (Wellferon, Alferon, and Betaferon) and ribavirin were administered [18]. This work warrants further in vivo investigation of the mentioned therapeutic agents.

## 4. Other therapies

### 4.1. Colchicine

Colchicine is an anti-inflammatory drug that exerts its effects by preventing microtubule polymerization [48], inhibiting leukocyte migration, and phagocytosis. Common adverse effects include diarrhea, nausea, vomiting, and abdominal pain. More rarely, it can cause hepatic necrosis, renal failure, disseminated intravascular coagulation, hair loss, bone marrow suppression, peripheral neuritis, and death [49].

#### COLCORONA Trial

The Montreal Health Institute is carrying out a large multi-center randomized controlled trial to investigate the role of colchicine in the treatment of COVID-19. The study estimates enrollment of 60,000 subjects, and a completion date of September 2020 has been suggested [19].

### 4.2. Glucocorticoids

Glucocorticoids act on glucocorticoid receptor elements (GRE) to exert their anti-inflammatory effects. They have a wide range of effects including but not limited to decreased production of inflammatory mediators, inhibition of the arachidonic acid pathway, and reduced migration of immune cells to the site of insult. Adverse side effects are numerous, including osteoporosis, avascular necrosis, weight gain, hypothalamic pituitary adrenal axis dysfunction, increased incidence of opportunistic infections, and psychosis.

#### 4.2.1. Early, Low-Dose and Short-Term Application of Corticosteroid Treatment in Patients with Severe COVID-19 Pneumonia: Single-Center Experience from Wuhan, China

This retrospective review of 46 patients included 26 patients who received intravenous methylprednisolone [20], as detailed in Table 1. There were no other notable differences in patient parameters. Clinical symptoms and CT chest results were compared before and after therapy. Of 46 patients, 27 (59%) were febrile and 15 (33%) received the drug.

For the methylprednisolone group, patients showed fever resolution at 2 ± 0.28 days, whereas, for the control group, the resolution of fever was at 4.39 ± 0.70 days. All 46 patients received oxygen therapy. Those who received methylprednisolone required supplemental oxygen for 8.2 (7–10.3) days, whereas the control group needed it for 13.5 (10.3–16) days. In patients who received methylprednisolone, an improvement in the absorption degree of focus was observed on CT. This trial’s limitations include its retrospective design, small sample size, and absence of mid- to long-term outcome measures.

#### 4.2.2. Clinical Evidence Does Not Support Corticosteroid Treatment for 2019-nCoV Lung Injury

In this review, the author provided a summary of the clinical data to this point on corticosteroid use in the treatment of SARS and MERS [21]. They suggested that the use of corticosteroids for SARS and MERS likely led to no benefit with evidence of definite harm in some cases. In the SARS case, there was a delayed clearance of viral RNA from blood, increased incidence of psychosis, diabetes, avascular necrosis, and osteoporosis associated with corticosteroid use. The authors concluded that COVID-19 is not likely to differ from MERS and SARS as far as response to corticosteroids. The decision to use glucocorticoids should be made after reviewing each patient’s situation’s risks and benefits.

#### 4.2.3. Effect of Dexamethasone in Hospitalized Patients with COVID-19-Preliminary Report (RECOVERY Trial)

The preliminary results of the dexamethasone arm of the RECOVERY trial were released due to clear benefits in the treatment. In this trial, 2104 patients were randomly allocated to the dexamethasone arm and 4321 patients to the usual care arm. In those who required mechanical ventilation or supplemental oxygen, a 28-day survival benefit was observed (35%, *p* < 0.001, and 20%, *p* < 0.002, respectively). No such benefit was observed in patients who did not require respiratory support. Given the wide accessibility of dexamethasone globally, this trial is promising that it shows a clear benefit to patients with the most severe illness [22].

## 5. Serotherapy

### 5.1. Convalescent Plasma (CP) Transfusion/Antibody Therapy

Convalescent plasma transfusion is a form of conferring the passive immunity of an individual who has had exposure to and cleared a certain microbe to an individual who is currently infected. The idea is that the antibodies in the donor’s serum will neutralize the virus in the recipient. This form of therapy was used centuries ago to treat infections before the development of antimicrobials and was also used with some success in the treatment of SARS-1 and MERS [50].

#### 5.1.1. The Feasibility of Convalescent Plasma Therapy in Severe COVID19 Patients: A Pilot Study

The potential efficacy of convalescent plasma therapy was explored through the transfusion of a single 200-mL dose of convalescent plasma from recently recovered donors to ten severely ill patients [23]. Following transfusion, clinical symptoms improved significantly. Increased oxyhemoglobin saturation was observed within three days. Observations included elevated lymphocyte count, decreased CRP, and an undetectable viral load was noted in seven patients with the previous viremia. No severe adverse events were observed. In all ten patients, fever, cough, shortness of breath, and chest pain disappeared or largely improved within 1–3 days of therapy. Before therapy, three patients were receiving mechanical ventilation, three high flow nasal cannular oxygen, and two received low flow nasal cannular oxygen. Two patients were weaned from mechanical ventilation to high flow nasal cannula, and one patient discontinued high flow nasal cannula. In one patient, conventional continuous nasal cannular oxygen was shifted to intermittent. Some of the patients who received the transfusion demonstrated improvement in CT with the absorption of lung lesions. A historical control was used for comparison. Of those who received convalescent plasma, three were discharged and seven improved significantly and were ready for discharge. In the control group, there were three deaths, six cases with a stable status, and one improvement. Limitations include small sample size, historical control, ill-defined endpoints, and absence of randomization or blinding.

#### 5.1.2. A Highly Conserved Cryptic Epitope in the Receptor-Binding Domains of SARS-CoV-2 and SARS-CoV

The authors demonstrated that a highly conserved epitope is common between SARS-CoV and SARS-CoV-2 [24]. In vitro studies did not demonstrate the total neutralization of SARS-CoV-2 with the SARS-CoV antibody CR3022 (convalescent plasma retrieved from a patient previously infected with SARS-CoV). While in vitro studies did not show perfect neutralization, the authors recommended in vivo experimentation given the different biochemical environment. They cited in vivo protection despite the absence of in vitro antibody neutralization of influenza virus, herpesvirus, cytomegalovirus, and dengue.

#### 5.1.3. Convalescent Plasma for COVID-19. A Randomized Clinical Trial

This study demonstrated that the administration of convalescent plasma to hospitalized patients might? be redundant, according to the authors [25]. Most of the enrolled patients had titers comparable to donors at baseline, questioning the rationale behind administering donor plasma to such individuals. The authors suggested that physicians should test for SARS-CoV-2 antibodies in hospitalized patients before treatment with convalescent plasma. With this, only those without antibodies would be treated. Further, the authors found no benefit in the experimental group survival rate, 15-day disease severity, or length of hospital stay for the trial duration. A major limitation of this trial in assessing the efficacy of convalescent plasma therapy is the inclusion of only hospitalized patients. It would be interesting to determine whether convalescent plasma therapy has a role to play in post-exposure prophylaxis or early infection treatment.

### 5.2. Tocilizumab

Tocilizumab is a recombinant human monoclonal antibody with activity as an interleukin-6 (IL-6) receptor inhibitor [51]. The function of IL-6 is multifaceted and is involved in numerous autoimmune disease processes. One of the main functions of IL-6 is to participate in the acute phase reaction in response to a biological insult. By inhibition of the IL-6 receptor, tocilizumab effectively restricts the biological role of IL-6 as a pro-inflammatory protein. In the context of COVID-19, tocilizumab may be useful in addressing the hyper-inflammatory state in patients with severe disease.

#### 5.2.1. Tocilizumab for Patients with COVID-19 Pneumonia

The TOCIVID-19 is a multicenter, single-arm, hypothesis-driven trial set out to determine the one-month lethality rate in those who receive tocilizumab versus those who receive the standard of care treatment [28]. Patients hospitalized with COVID-19 were enrolled if they had oxygen saturation lower than 93% on ambient air, were receiving supplemental oxygen, or were mechanically ventilated. Experimental lethality rated was compared to the null-hypothesis rates of 20% and 35% for 14- and 30-day lethality, respectively. The authors also noted that patients who received corticosteroids concurrently demonstrated a survival benefit.

This study is limited by its methodology. Notably, the absence of a robust control makes establishing efficacy difficult. Therefore, tocilizumab may be effective, but more robust trials are necessary to make that determination.

#### 5.2.2. Tocilizumab for Treatment of Mechanically Ventilated Patients with COVID-19

This trial included 154 COVID-19 patients (78 tocilizumab, standard of care) who required mechanical ventilation [29]. The primary endpoint was the probability of survival following intubation. The authors assert that the baseline characters were similar in the two groups. However, there was a five-year difference in mean age (lower in tocilizumab group), chronic pulmonary disease (10% tocilizumab vs. 28% control), and a lower averaged D-dimer level. In this group, the authors concluded that tocilizumab was associated with a decreased risk of death (45% reduction) and called for the initiation of randomized control trials. We echo the need for randomized control trials but question the strength of the association due to the difference in age, chronic pulmonary disease, and D-dimer levels between the two groups. Those are likely to be significant confounders.

#### 5.2.3. Comparative Survival Analysis of Immunomodulatory Therapy for COVID-19 “Cytokine Storm”: A Retrospective Observational Cohort Study

This retrospective analysis of electronic health records included 3098 hospitalized COVID-19 patients who met the criteria for cytokine storm (defined as ferritin >700 ng/mL, CRP >30 mg/dL, or lactate dehydrogenase >300 U/L) [30]. The authors suggested that tocilizumab alongside corticosteroids and corticosteroids alone provide a survival benefit compared to standard of care. They identified that there are innate limitations due to the retrospective design and variance among health systems from where the data were derived.

## 6. Anticoagulant and Recombinant Human Soluble ACE2 Therapies

The primary agent used for the treatment of infection-associated coagulopathy is heparin (specifically, low molecular weight heparin (LMWH)). Heparins work through potentiation of anti-thrombin effects, which leads to the inactivation of thrombin, Factor Xa, and Factor IXa [52]. This effect ultimately inhibits the coagulation cascade. The primary adverse event associated with heparin use is increased risk of bleeding and, more rarely, heparin-induced thrombocytopenia [53]. Mortality in those with COVID-19 was shown to be related to elevated D-dimer and prothrombin and decreased platelet count, indicating possible disseminated coagulation [27]. Thus, anticoagulation may remedy this pathology and improve survival.

Angiotensin-converting enzyme 2 is a membrane-bound protein that is the molecular site of target and entry of SARS-CoV-2 [54]. Theoretically, introducing recombinant human soluble ACE2 may neutralize the virus before it can attach to the membrane-bound ACE2, thus preventing infection.

### 6.1. Inhibition of SARS-CoV-2 Infections in Engineered Human Tissues Using Clinical-Grade Soluble Human ACE2

This in vitro study demonstrates that SARS-CoV-2 is capable of infecting human blood vessel and kidney organoids [26]. In addition, the inhibition of SARS-CoV-2 infection of human kidney and blood vessel organoids with the use of human recombinant soluble ACE2 was shown. Limitations include the omission of the most significant infection site: the lungs. Further, this study focused on the early stage of infection. Further investigation (in vivo and/or in vitro) is required to establish safety and efficacy as well as its function in later stages of infection. It also provides some insight into pathophysiology that may involve coagulation. Further investigation into the pathology that follows infection of blood vessels and kidneys with SARS-CoV-2 is of importance. Elevated D-dimer has been seen in patients with COVID-19, and it is not unreasonable to suggest that there may be a link with a virus infection site [27].

### 6.2. Anticoagulant Treatment Is Associated with Decreased Mortality in Severe Coronavirus Disease 2019 Patients with Coagulopathy

This retrospective study analyzed the outcomes of 449 consecutive patients hospitalized for COVID-19 [27]. Ninety-nine (22%) of these patients received heparin for seven or more days. D-dimer and prothrombin were shown to be positively associated with 28-day mortality, while the platelet count was negatively correlated. Generally, no difference was noted in the 28-day mortality of patients with or without heparin (30.3% vs. 29.7%). However, the 28-day mortality of those who received heparin compared to those who did not was lower in patients who had a sepsis-induced coagulopathy (SIC) score of 4 or more (40.0% vs. 64.2% mortality) or a D-dimer >6 times upper limit normal (32.8% vs. 52.4% mortality). All patients received antiviral therapy concurrently. The author concluded that there is evidence of benefit to using LMWH in those with SIC >4 and elevated D-dimer. Limitations include retrospective design, and lack of information on risk stratification of patients. For example, those who were sicker may have been more likely to receive heparin.

## 7. Conclusions

At the time of writing, there is not enough evidence to determine whether any therapeutic agent has strong efficacy in the treatment of COVID-19. Notably, many of the reviewed trials lack the robust methodology required to make a sound conclusion. For example, the sample size is severely limited in several studies, reducing the power of the already weak outcomes these trials have produced. Further, the absence or the use of inadequate control methods limits the trials’ capacity to establish efficacy. The inclusion of a robust control group is essential to determine whether there has been a difference in the trajectory of the disease due to the therapeutic agent. With that said, the rate at which the literature is evolving is rapidly increasing, and new potential therapeutics are being identified.

Depending on the therapeutic agent, its efficacy may be at different points in the natural history of the disease. For example, convalescent plasma therapy does not appear to be efficacious in hospitalized COVID-19 patients because those patients have already produced antibodies. This is not to say that there is no role for personal-centered therapy early on in disease or prophylaxis—this remains to be explored.

The preliminary results of the RECOVERY trial outline a clear role for dexamethasone in treating COVID-19 in those requiring respiratory support. The data are robust, the effect size is large, and the cost is low. This is extremely promising, and we feel comfortable recommending the use of dexamethasone in the population outlined in the RECOVERY trial (in those requiring respiratory support). It is important to note that there is no survival benefit for those not requiring respiratory support, and treatment should be offered judiciously.

The research surrounding the efficacy of remdesivir is still evolving, and numerous agencies are running randomized control trials. Data from the ACTT-1 trial demonstrate a decrease in illness duration by approximately four days compared to placebo. Trials currently underway to watch are the SIMPLE-Severe and SIMPLE-Moderate trials, which explore the potential benefits of remdesivir in those with severe and moderate disease, respectively. Given there is no clear mortality benefit demonstrated at this time, the decision to use remdesivir should be made carefully and within emergency use guidelines.

We outline several therapeutics at various stages of development. Most, if not all, of these therapeutics still require a considerable level of investigation to establish efficacy and determine adverse effect profiles. The widespread use of any of these agents is not yet recommended, although exceptions have been made in various regions surrounding compassionate care or emergency use. However, we recommend monitoring existing comparative trials to guide clinical decision-making and initiating new trials with these agents where appropriate. 

We want to highlight the importance of avoiding the recommendation of unproven therapies to limit the harm that may result from the use of these agents.

## Figures and Tables

**Table 1:** Description of the studies using different types of therapeutic agents.

Therapeutic Agent	Methodology	Key Findings	Reference
Chemotherapy			
Hydroxychloroquine +/− Azithromycin	*n* = 64 Study: double blind randomized control trial Treatment: HCQ 400 mg/day × 5 days	Improved total time to recovery, resolution of fever, cough remission, and pneumonia severity	[5]
	*n* = 36 Study: preliminary trial Treatment: HCQ 200 mg TID for 10 days	Showed large reduction in viral carriage following treatment with HCQ and HCQ + Az	[6]
	*n* = 80 Study: single hospital in-patients, non-randomized, no control, no blinding Treatment: HCQ 200 mg TID for 10 days Az 500 mg OD (Day 1), 250 mg OD (Days 2–5)	93% patients negative viral RNA PCR by day 8. 97.5% negative viral cultures by Day 5. 81% of patients had mild disease; initiation to discharged mean 4.1 days	[7]
	*n* = 11 Numerous comorbidities Treatment: HCQ 600 mg/day for 10 days, Az 500 mg OD (Day 1), 250 mg OD (Days 2–5)	5–6 days post treatment, 8/10 nasal swabs positive. One patient death, two transferred to ICU. Discontinuation due to QTc prolongation in 1 patient	[8]
	*n* = 84 Consecutive admissions, retrospective trial	11% of patients developed a QTc >500 (high risk for arrhythmia). 30% demonstrated a QTc increase > 40 ms	[9]
Antivirals			
Lopinavir/Ritonavir (LPV/R)	*n* = 199 Study: open label randomized control trial LPV: 400 mg/day R: 100 mg/day × 14 days	No significant improvement in mortality or viral load. No benefit compared to standard care	[10]
	*n* = 120 Study: Retrospective trial of admitted patients in Wuhan LP: 400 mg/day R: 100 mg/day	Lack of treatment with LPV/R is associated with an increase in duration of viral shedding vs. control. Old age is associated with an increase. Benefits of LPV/R present when treated <10 days after symptom onset	[11]
+Abridol	*n* = 44 Study: exploratory randomized control trial LPV: 400 mg/day R: 100 mg/day Arbidol: 600 mg/day × 7–14 days	No significant difference in time to viral conversion from positive to negative. 24% of LPV/R group experienced adverse effects	[12]
Remdesivir	Study: Investigation of in vitro activity of chloroquine and remdesivir	Remdesivir: 1.76 µmol/L for EC90 in nonhuman primates Chloroquine: 6.09 µmol/L for EC90 in non-human primates	[13]
	*n* = 18 Study: Rhesus macaques drug vehicle control, prophylactic vs. therapeutic. Inoculated with MERS-CoV	When given prophylactically, prevents infection. When given therapeutically, leads to clinical improvement. Decreased number and severity of lung lesions, reduced viral replication in the lungs	[14]
	Single case study	Patient showed improvement within a day of remdesivir treatment. Oropharyngeal swab converted to negative, nasopharyngeal remained positive	[15]
	*n* = 12 Study: 3/12 patients received remdesivir under compassionate use. Treatment: 200 mg IV first day, then 100 mg IV once daily	Mild GI symptoms, transient aminotransferase elevations. One episode of bloody stool	[16]
Clinical trial in progressPhase 3	*n* = 1063 Study: multi-center randomized control trial	Improved time to recovery (11 vs. 15 days, *p* < 0.001), mortality (8% vs. 11.6, *p* = 0.059). Modest effect size	[17]
Oseltamivir/Amantadine	In vitro investigation of the anti-SARS-CoV activity of numerous approved drugs	No inhibition of SARS-CoV-2 cytopathic effect with use of oseltamivir or amantadine in vitro	[18]
Colchicine			
Clinical trial in progressPhase 3	*n* = 6000 Study: Multi center, double blinded randomized control trial	N/A	[19]
Glucocorticoids			
	*n* = 46 Study: retrospective review, methylprednisolone 1–2 mg/kg/day 5–7 days	Improvement in time to resolution of fever, duration of supplemental oxygen use, and CT absorption degree of focus	[20]
	Review of literature surrounding corticosteroid use during SARS and MERS	Corticosteroids use for SARS and MERS showed no benefit, but harming in some cases	[21]
Recovery Trial	*n* = 2104 (dexamethasone) Study: open-label randomized controlled trial	In patients with mechanical ventilation, dexamethasone reduced mortality by 1/3, and by 1/5 in those receiving oxygen alone. No survival benefit for those not requiring respiratory support	[22]
Convalescent plasma			
	*n* = 10 severely ill patients Treatment: 200 mL IV	In all 10 patients, fever, cough, shortness of breath, and chest pain disappeared or largely improved within 1–3 days of therapy initiation	[23]
	In vitro study determining the activity of convalescent plasma from a recovered SARS-1 patient against SARS-CoV-2	Demonstrates conserved epitope in SARS-1 and SARS CoV-2. Viral inhibition of SARS-CoV-2 with specific biochemical configuration	[24]
	Study: Randomized control trial	Trial halted early due to 53/66 patients having anti SARS-CoV-2 antibodies at baseline. No difference in mortality, severity, or duration of hospital stay was observed over 15 days	[25]
Anticoagulants			
Heparin	In vitro study exploring pathophysiology of SARS-CoV-2 infection. Human blood vessel and kidney cell organoids	Demonstrates ability for SARS-CoV-2 to infect blood vessel and kidney organoids	[26]
	*n* = 449 Study: stratification based on risk level for coagulopathy. *n* = 94 enoxaparin 40–60 mg/day, *n* = 5 unfractionated heparin 10,000 U/day	Demonstrates benefit for those with sepsis induced coagulopathy scores ≥4 or D-dimer >6 × ULN	[27]
*Recombinant Human Soluble Angiotensin Converting Enzyme 2 (rhsACE2)*			
	In vitro study exploring pathophysiology of SARS-CoV-2 infection. Human blood vessel and kidney cell organoids	Rates of infection of blood vessel and kidney organoids were reduced compared to controls in the presence of recombinant human serum ACE2	[26]
*Biological Treatment-Tocilizumab*			
TOCIVID-19	*n* = 301 Study: a multicenter, single arm trial Hypothesis driven – null = 20 and 35% lethality rate at 14 and 30 days	Tocilizumab reduced lethality rate at the 30-day interval (22.4%), rejected null hypothesis (35%). *p* ≤ 0.001. Null hypothesis not rejected for 14-day interval. Suggest use of tocilizumab awaiting phase 3 trials	[28]
	*n*= 154 Study: single center observational cohort study	45% reduction in hazard of death in those who received tocilizumab vs. untreated. Increased risk of superinfection with tocilizumab (54% vs. 26%), but no difference in case fatality due to superinfection	[29]
	*n*= 3098 Study: retrospective analysis of health records. COVID-19 hospitalizations with cytokine storm over a month and a half period	The use of corticosteroids alone, or in conjunction with tocilizumab improved hospital survival compared to standard care (no immunomodulatory medication) alone	[30]

HCQ, hydroxychloroquine; Az, azithromycin; QTc, corrected QT interval; LPV, lopinavir; R, ritonavir; ULN, upper limit of normal.
